# Spatial-temporal variability of methane fluxes in lakes varying in latitude, area, and depth

**DOI:** 10.1016/j.heliyon.2023.e18411

**Published:** 2023-07-21

**Authors:** Lingling Li, Bin Xue

**Affiliations:** a School of Geography Science, Jiangsu Second Normal University, Nanjing, China; b State Key Laboratory of Lake Science and Environment, Nanjing Institute of Geography and Limnology, Chinese Academy of Sciences, Nanjing, China

**Keywords:** Greenhouse gas, Global lake, Spatial heterogeneity, Seasonal variability

## Abstract

Many previous studies have found spatial and seasonal variabilities in CH_4_ fluxes, which could significantly affect lake-wide CH_4_ budgets. However, the ways in which the spatial and seasonal patterns of CH_4_ fluxes vary among lakes on a global scale is largely unknown. We compiled literature on CH_4_ flux data from global lakes and analyzed the spatial and seasonal variabilities for lakes varying in latitude, maximum depth, and area. Spatially, we found a significant linear relationship between the ratio of littoral to profundal fluxes and lake morphology (more related to area than depth), while globally, half of the lakes would have within 5% error of CH_4_ emission estimation under single-zone sampling. Seasonally, mid-latitude lakes showed higher CH_4_ fluxes in the summer and autumn, indicating the influence of temperature and autumn overturn, and the latter being largely related to maximum depth. Globally, due to abundant shallow lakes in the mid-latitude zone, approximately 99% of lakes had higher fluxes in the summer, while 75% of lakes showed errors in CH_4_ emission estimation within 20% when only the summer flux was investigated. In the high-latitude lakes, CH_4_ evasion during the spring ice-off period was significantly correlated with lake maximum depth, while lake area was also important when analyzing the CH_4_ diffusive flux. Our study yields preliminary conclusions about spatial and seasonal patterns of CH_4_ flux in different lake types, which are fundamental to building an effective sampling strategy and to determining an accurate CH_4_ budget from global lakes.

## 1 Introduction

CH_4_ is the second most impactful greenhouse gas after CO_2_, and on a 100-year time horizon, the potential warming effect of CH_4_ is approximately 28 times that of CO_2_ [1]. The atmospheric CH_4_ concentration has dramatically increased since the 1750s, and after stagnation from 2002 to 2006, it started to increase again in 2007 [2]. The accurate evaluation of the global CH_4_ budget is important for developing realistic approaches to mitigate climate change. Freshwater is recognized as an important natural source of CH_4_, although it covers a small fraction of the Earth's surface area [[3], [4], [5]], and lakes are estimated to contribute approximately 62% of freshwater CH_4_ emissions [6]. However, few previous estimations of global lake CH_4_ emissions have included the spatial and seasonal variability in CH_4_ fluxes, which are considered important for lake-wide CH_4_ budgets [[7], [8], [9]].

Spatially, single point or regional measurements are insufficient to accurately estimate lake-wide CH_4_ fluxes into the atmosphere [[8], [9], [10]]. It is assumed that the littoral zone contributes more than 60% of CH_4_ emissions in shallow lakes [11]. In contrast, the small (0.24 km^2^) and deep (maximum depth >10 m) Lake Gerzensee had higher CH_4_ fluxes in the central region [12]. Hence, the spatial CH_4_ fluxes may be different for lakes varying in area and depth. Furthermore, significant seasonal variations in CH_4_ fluxes were also found to be accompanied by different seasonal temperature and water environment conditions [9,13,14]. Hence, the CH_4_ flux may have different seasonal patterns in lakes in different latitude zones. In summary, we hypothesized that the spatial and seasonal variability in the CH_4_ flux would show different patterns for lakes varying in latitude, area, and depth. Hence, we compiled direct measurements of spatial and seasonal CH_4_ fluxes (including diffusion and ebullition) from lakes worldwide, and the spatial and seasonal CH_4_ flux variability patterns were analyzed for different lake types.

## 2 Materials and methods

### 2.1 Collection of published data

The areal lake CH_4_ fluxes and concentrations, including spatial or seasonal sampling, were collected from the literature. Reservoirs were excluded owing to the specific gas pathways in the dam [15], as well as saline or brackish lakes due to their unique biogeochemistry and paucity of direct measurements. The spatial heterogeneity in CH_4_ fluxes was analyzed for 38 lakes with sampling sites distributed both in the littoral (nearshore) and profundal (open water) zones, while only 33 lakes explicated both the area and maximum depth data in the literature, and 41 and 40 lakes were used for area and maximum depth analysis, respectively. A total of 125 lakes, with 13, 28 and 84 lakes in low-, mid- and high-latitude areas, respectively, were selected with sampling in both the high CH_4_ flux and low CH_4_ flux seasons to study seasonal variability (Supplementary Table S1). The CH_4_ fluxes found in the literature were mainly measured with a static or floating chamber technique, in which the chambers without bubble shields received CH_4_ by both diffusion and ebullition, and the surface-water CH_4_ concentrations were measured using the headspace equilibrium technique [3]. Moreover, if a study clearly stated that the chamber was used only to measure CH_4_ diffusive flux, the data were also considered to represent CH_4_ diffusive flux in this study. CH_4_ diffusive flux was also calculated with a boundary layer diffusion model (Fick's law) in some studies (e.g., Ref. [8]).

### 2.2 Spatial CH_4_ flux

The 38 lakes with available spatial CH_4_ data were divided into littoral and profundal zones according to the description in each study. In each lake, CH_4_ fluxes from the same lake zones were averaged if more than one value was available. The ratio of littoral to profundal CH_4_ flux (L/P) was proposed to quantify the spatial variability in CH_4_ flux. Note that the L/P of 7 deep and large lakes were calculated via CH_4_ diffusive flux or surface-water CH_4_ concentration because of a lack of CH_4_ ebullition data. Because the data of maximum depth was more frequently offered in the collected literature than mean depth, maximum depth rather than mean depth was used in this study. In addition, to simultaneously reflect lake area and depth, the ratio between lake area (km^2^) and maximum depth (m) (Area/MaxDepth) was calculated as one of the lake physical parameters. We performed linear, polynomial, and smooth spline regressions to evaluate the relationship between L/P and lake physical characteristics and evaluated quadratic relationships for piecewise regressions.

To assess the representativeness of sampling sites at the lake center, we assumed that the lakes had similar areas of littoral, intermediate and profundal zones, that is, each lake zone equals 1/3 of the lake area, and the CH_4_ flux from the intermediate zone was the average of the CH_4_ flux in the profundal and littoral zones. According to the calculated L/P, if we hypothesize that the CH_4_ flux in the profundal zone is x, then the CH_4_ flux in the littoral zone is L/P*x, and the CH_4_ flux in the intermediate zone is (x + L/P*x)/2. Then, whole lake CH_4_ emissions estimated with both the littoral, intermediate and profundal fluxes are calculated as follows:

(1) Em=(1/3*x+1/3*x*L/P+1/3*((x+L/P*x)/2)))*area

when only the profundal flux was used to represent the whole lake, CH4 emissions could be estimated as Ep = x * area. Correspondingly, when using only the littoral zone flux, CH4 emissions were estimated to be El = x*L/P*area. We calculated the ratio of Ep/Em and El/Em to evaluate the error of CH_4_ emission estimation. Please see Table S3 for the calculation processes.

### 2.3 Seasonal CH_4_ flux

To analyze seasonal CH_4_ flux from the 152 lakes, the sampling dates were divided into spring/autumn (March to May), summer/winter (June to August), autumn/spring (September to November) and winter/summer (December to February) in the Northern/Southern Hemisphere, except for lakes in tropical areas. The tropical climate shows no significant temperature differences but is characterized by higher rainfall in summer [16,17]. Tropical lakes may be flooded by river inflow or local rainfall or by both; thus, the high-water period may be delayed for months after summer rain [18]. Hence, the sampling period of tropical lakes was separated into low- and high-water seasons according to the regional climate and the timing of inundation.

For analysis of the seasonal CH_4_ flux at different latitudes, the studied lakes were divided into low-latitude (<24°), mid-latitude (24°–54°), and high-latitude (>54°N) zones. The mid-latitude region included mainly subtropical and temperate climates, which had obvious seasonal temperature variability. For mid-latitude lakes, higher CH_4_ emissions probably occur in summer (e.g., Ref. [10]) or during the lake turnover period in spring or autumn [19], and sampling for CH_4_ more frequently occurs in summer and autumn according to our collected data. Therefore, to assess the representativeness of limited seasonal sampling, we hypothesize that the days for each season are equivalent. Then, we calculated the ratios of whole-year CH_4_ emission estimation with seasonal flux in summer (Es), autumn (Ea) or both (Esa) to estimated CH_4_ emission using the seasonal mean flux (Esm). In addition, the ratio of CH_4_ flux in summer to that in autumn (Fsummer/Fautumn) was used to analysis the seasonal difference between summer and autumn.

Lakes at high latitudes usually had ice cover during the winter, although a small part of the gas could escape through openings in the ice owing to ice cracks caused by increased pressure and/or warm waters from inflows and frequent CH_4_ bubbling [20] or from vegetation emergence through the ice [21]; most of the CH_4_ was trapped in the ice and underlying water when the ice was present. If CH_4_ escapes oxidation under ice, CH_4_ accumulation under the ice could result in gas “storage”, and large diffusion emissions occur when the ice melts in spring [[22], [23], [24]]. Hence, we focused on the contribution of CH_4_ flux during the ice melt and a comparison with that in the ice-free period. Ice-melt refers to springtime when ice starts to thaw until complete ice-off; the ice-free period includes summer and the possible autumn turnover period. Because of the large differences in the duration dates of the two periods, for example, complete thaw could take only two weeks in northern Swenden [25], the CH_4_ fluxes were presented as the summary during each period, that is, the mean flux in each period multiplied by its duration days (unit of mmol per m^2^). Previous studies estimated the spring efflux by using bubble trapped under ice or floating chambers, as well as by sampling CH_4_ dissolved in lake water immediately before and after ice melt or by monitoring CH_4_ fluxes with eddy covariance [25]. Referring to the method of [26], we calculated the percentage contribution of ice-melt flux to annual CH_4_ emission to reduce the uncertainty between different methods used to estimate CH_4_ fluxes.

Considering the different seasonal lake environments, which result in various CH_4_ emission modes, the seasonal CH_4_ flux was analyzed separately among the three latitude regions. We used the Kruskal‒Wallis test to compare CH_4_ fluxes among the four different seasons and a Dunn-Bonferroni test for post hoc comparisons. The Mann‒Whitney *U* test was conducted to compare CH_4_ fluxes in the two tropical seasons. Pearson correlation and linear regression analysis were used to analyze the relationship between seasonal CH_4_ flux and lake physical characteristics.

### 2.4 Spatial and seasonal variability in global lakes

Robust models to investigate the relationship between spatial and seasonal CH_4_ fluxes and lake physical characteristics were chosen to estimate L/P or the seasonal proxy indicators (only for mid-latitude lakes) for global lakes. Lake depth data were excluded in most of the datasets on lake characteristics at the global scale [27,28], except the HydroLAKES dataset [29], which includes mean lake depth, and the Global Lake Database (GLDBv2), which includes hiatus mean and maximum depth data [30]. We developed the Fresh-Water Lake-Depth Dataset (FWLDDS) in GLDBv2 with data mostly from Wikipedia except for Chinese lakes, which were referenced from the recently created lake survey database [31]. The lakes for which at least lake area and mean or maximum depth data were available were included from the FWLDDS, and the final dataset had 5762 lakes (Supplementary Table S2). Since approximately 20% of lakes have only mean depth and lack maximum depth data, which is a key factor for predicting CH_4_ flux, a linear regression model was run to predict maximum depth based on lake area and mean depth; the simulation value was significantly correlated with the measured value, suggesting the reliability of this model (Function 2).

(2) $$\mathrm{Max}\;depth=2.045\times mean\;depth+0.003\times area\;\left(adj\;R^{2}=0.90,df=4382,p<0.0001\right)$$

here, lake area is in km^2^, and maximum and mean depth are in m. Then, maximum depth data were supplemented for the HydroLAKES dataset using Function 2 based on lake mean depth and area [29].

## 3 Results and discussion

### 3.1 Spatial flux patterns

**Fig. 1:**
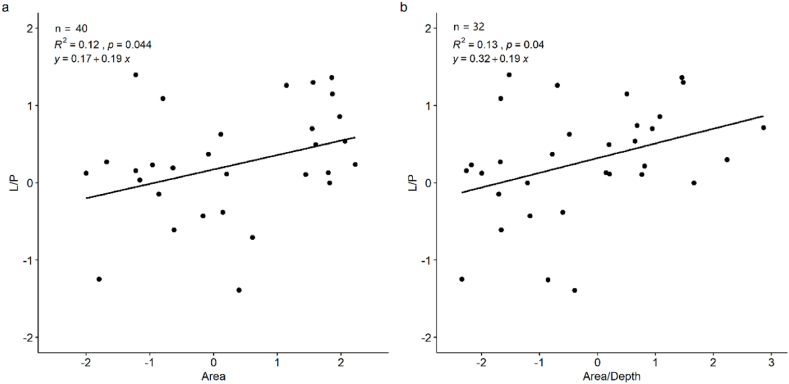
The ratio between CH_4_ emissions in the littoral and profundal zones (L/P) in relation to lake area (a) and the ratio between lake area and maximum depth (MaxDepth) (b). The units for lake area and MaxDepth were km^2^ and m, respectively, and all the data were natural log transformed.

The spatial variability in the CH_4_ flux had different characteristics among the various lake types. Limited by data size, although we tried multiple regression models, only the linear models between lake area, Area/MaxDepth and L/P were statistically significant with *p* < 0.05 but with relatively low R^2^ (Fig. 1a & b). L/P showed a significant correlation with lake area and Area/MaxDepth, rather than maximum depth. Most lakes had higher CH_4_ fluxes in the littoral zone, while higher CH_4_ fluxes in the profundal zone could exist only in some, not all, small and deep lakes (see data in Supplementary Table S1). Typically, the littoral zone has abundant available organic matter in sediments originating from high productivity or influx from the watershed [9,14,32], and the increased methanogenesis rates in the littoral zone generates greater CH_4_ storage during summer stratification and enhanced CH_4_ emission to the atmosphere by bubbling [33]. In contrast, without abrupt water depth variation in shallow lakes, the lake currents are determined mostly by the prevailing wind direction and lake morphology, inducing erosion in the profundal zone and deposition along the shoreline and in the lake bays. The profundal sediment in large and shallow lakes may be susceptible to erosion by lake currents, inducing lower CH_4_ production, such as that in Lake Taihu, which has little soft sediment in the profundal zone [14,34,35].

Specifically, we found that L/P and Area/MaxDepth showed significant positive correlations, and when the Area/MaxDepth >1, L/P > 1 (Fig. 1b), suggesting that when the lakes tend to be larger and shallower, that is, with less lakebed slope, their CH_4_ shows higher flux in the littoral zone than in the profundal zone. Håkanson (1977) [36] described a linear relationship between slope and sediment water content, which had a direct link to sediment bulk density and grain size. In addition, topography-induced water depth variations may influence the lengths of internal waves at the bottom sediment, thereby directly affecting the bottom water currents and material transmission. The steep slopes of small and deep lakes facilitate sediment migration to profundal regions, resulting in a higher proportion of easily degradable organic matter (e.g., algae) in central sediments than in the near-shore zone [16,37]. In addition, the strong seasonal stratification of deep lakes contributes to the low dissolved oxygen concentrations or anoxic conditions in deep waters [16,19,38], leading to high CH_4_ production and effusion.

However, CH_4_ produced in the deeper sediment is at a greater risk of oxidation in the sediment and water column, even for ebullition. A bubble model for Lake Stechlin shows that approximately 80% of CH_4_ trapped in the bubbles released from the nearly 70 m deep sediment would dissolve in the hypolimnion (>40 m depth) and leave little CH_4_ in the gas bubbles when reaching the surface, and less than 20% of the CH_4_ mass fraction remains in the bubbles released from 40 m deep sediment when reaching the surface [39]. On the other hand, oxic CH_4_ production is considered an important CH_4_ source correlated to sediment area and surface mixed layer volume [[40], [41], [42], [43]]. The CH_4_ concentration in the profundal zone of the large and deep lake is presumed to be determined more by oxic CH_4_ production than by anoxic CH_4_ production in the sediment [44]. Therefore, a deeper maximum depth may induce higher CH_4_ production but also increase CH_4_ oxidation in the profundal area, which is related to topography, trophic status, etc., while oxic CH_4_ production may also matter, especially in the stagnation period. All the above information explains why not all small and deep lakes show higher CH_4_ fluxes in the profundal zone.

Overall, the bottom topography, which could be reflected by the collocation of lake depth and area, has an influence on the deposition rate and sediment features, which are related to the spatial heterogeneity of sediment organic matter content and methanogenesis. Thus, we deem that the lake area and maximum depth should both be considered to estimate CH_4_ flux spatial variability, and the linear regression model between Area/MaxDepth and L/P was used for further analysis (Fig. 1b). Nonetheless, apart from limited training data, the relatively weak correlation coefficients between L/P and lake physical characteristics may also indicate the influence of other factors, such as lake eutrophication and allochthonous carbon input from river inflow. Lake eutrophication could be a factor for high CH_4_ flux in the profundal zone of deep lakes [38], and oxic CH_4_ production in the surface water may also increase with the improvement of productivity, and phytoplankton especially diatoms, cyanobacteria, green algae, and cryptophytes [45,46]. On the other hand, river inflow may increase the organic matter supply to the littoral zone, resulting in high CH_4_ emissions [47], and rivers could also directly bring dissolved CH_4_ into the lake, which may enhance or weaken CH_4_ flux spatial heterogeneity [8,23,48,49].

**Fig. 2:**
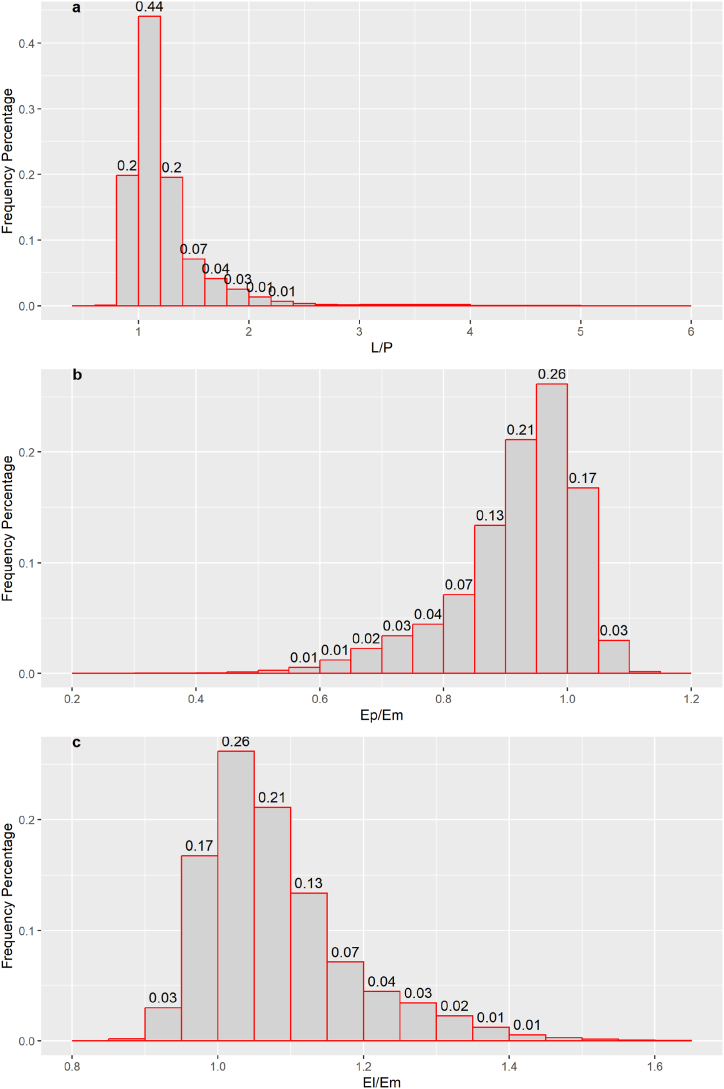
Frequency percentage of L/P, Ep/Em, and El/Em; Ep represents the estimated whole lake CH_4_ emission using CH_4_ flux in the profundal zone, Em represents the estimated CH_4_ emission using lake water mean CH_4_ flux, and El represents the estimated lake CH_4_ emission using CH_4_ flux in the littoral zone. The data on the bar are the percentage of the frequency, which is calculated as the ratio of frequency in each class to the total amount of data. Please see section 2.3 for the calculation process.

The estimated L/P for global lakes ranged from 0.50 to 6.30, with a mean value of 1.21 and a median value of 1.12. Generally, approximately 20% of lakes showed L/P < 1, and 64% of lakes had L/P values ranging from 1 to 1.4 (Fig. 2a). Correspondingly, only approximately 20% of lakes had Ep/Em > 1, and 20% of lakes had El/Em < 1. Almost half of the lakes showed Ep/Em ranging from 0.9 to 1.0, and El/Em ranged from 1.0 to 1.1 (Fig. 2b and c), indicating that half of the lakes showed errors of within 5% when using the littoral or profundal CH_4_ flux for whole lake CH_4_ emission estimation. That is, relatively low estimation error could occur for half of the global lakes when sampling only in littoral or profundal zones. However, considerable spatial sampling design should be conducted for relatively large or deep lakes.

### 3.2 Seasonal flux patterns

#### 3.2.1 Low-latitude lakes

**Fig. 3:**
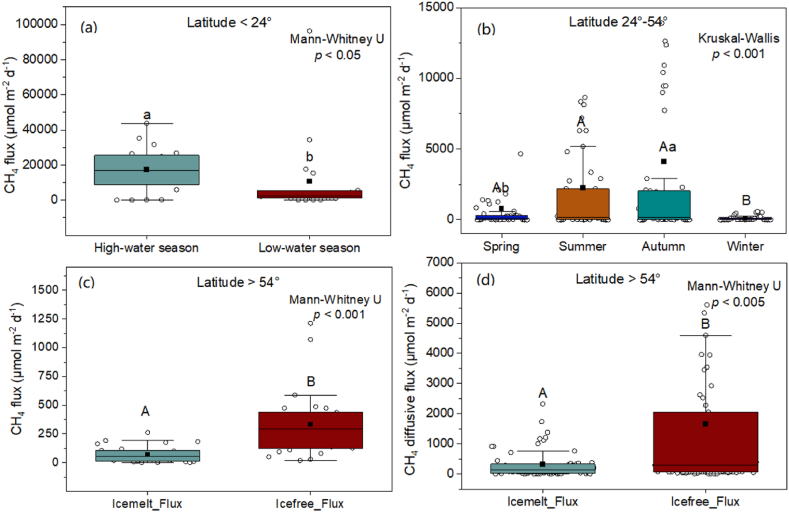
Seasonal variability in the total CH_4_ fluxes of lakes in different climatic zones and significant seasonal diffusive CH_4_ flux variability in high-latitude lakes. “■” shows the mean value. Letters above are comparison tests, values with different letters indicate significant differences, and different uppercase and lowercase letters show significant differences of *p* < 0.01 and *p* < 0.05, respectively.

A total of 13 lakes with CH_4_ flux measurements in both the high-water and low-water seasons were used for further analysis. Overall, low-latitude lakes had significantly higher CH_4_ fluxes in the high-water season than in the low-water season (*p* < 0.05, Fig. 3a). Ten out of the 13 lakes in Brazil showed inconsistent conclusions. Early studies using the static chamber technique indicated approximately 34 and 4 times higher CH_4_ fluxes in the high-water season than in the low-water season in the southern Pantanal [18] and Amazon [16], Brazil, respectively. The research by Marani and Alvalá (2007) [17] in the Pantanal region found that the diffusive flux showed a slight but not statistically significant seasonal variation, and the CH_4_ ebullition was variable but had no seasonal pattern. Notably, studies in the lakes of low-latitude areas with both CH_4_ diffusion and ebullition quantified concluded that most of the total CH_4_ emissions were ebullitive (more than 90%) [17,50]. Hence, more research that includes observations of CH_4_ ebullition is still needed to determine whether higher CH_4_ fluxes in the high-water season are valid across global low-latitude lakes.

CH_4_ ebullition is episodic and dependent upon many factors. CH_4_ bubbling from the sediment is considered to correlate with the shear stress in sediments caused by bottom currents, which in turn are influenced by wind, internal pressure gradients, and lake bathymetry [51]. Rising CH_4_ bubbles can dissolve in the water column and be stored or oxidized, and water depth and temperature are two important factors controlling the transfer processes of bubbles [52]. Hence, the season with relatively high temperature but low water level may have high CH_4_ ebullition flux per area, while high water level may cause anoxia in the sediment, resulting in high CH_4_ production and low CH_4_ oxidation. Therefore, to determine the seasonal CH_4_ flux in low-latitude lakes, the high variability in lake depth and area must be considered, and more regional research is needed to analyze their influence on CH_4_.

In low-latitude regions, other methods, such as satellite measurements, are complementary to the surface network of CH_4_. The study by Tunnicliffe et al., 2020 [53] using a high-resolution regional inversion framework coupled with satellite measurements of CH_4_ found that the Amazon and Pantanal wetlands both showed higher CH_4_ emissions in the high-water season. In addition, the estimation of CH_4_ emissions in the Pantanal region through regularly measured vertical atmospheric profiles revealed a large amount of CH_4_ emissions during rising water levels [54]. Hence, for the whole wetland area, higher CH_4_ emissions may be prevalent in the high-water season, while the situation in lakes is still ambiguous.

#### 3.2.2 Mid-latitude lakes

To analyze seasonal patterns, a total of 28 lakes with CH_4_ flux data in at least two seasons were chosen, among which 8 lakes were studied only in the summer and autumn. The CH_4_ flux in mid-latitude lakes showed significant seasonal variability, with the lowest flux in winter (*p* < 0.005) and higher fluxes in summer and autumn than in spring (*p* < 0.05) (Fig. 3b). Seasonally, temperature is usually considered an important factor influencing CH_4_ production and oxidation in mid-latitude lakes [55]. In the warm season, lakes may have higher sediment CH_4_ production under high temperature in the sediment and the supply of newly produced autochthonous organic matter [56]. In addition, the more serious eutrophication in the warm season could inhibit CH_4_ aerobic oxidation by eutrophication-created anoxic conditions [57,58]. Generally, temperature and trophic status are two main factors determining seasonal CH_4_ fluxes in mid-latitude shallow lakes [9,13,56,59].

However, our results showed that the autumn CH_4_ flux approached the level in summer, even with higher mean values but larger dispersion (Fig. 3b). Except for Ea/Esm, the seasonal CH_4_ flux proxies (i.e., Es/Esm, Esa/Esm and Fsummer/Fautumn) all showed negative but greater correlations with maximum depth than with lake area. The significantly high CH_4_ flux in autumn is likely to occur in deep lakes. For most deep lakes in the mid-latitude zone, summer stratification is a process in which much CH_4_ accumulates in anoxic deep waters. Then, the large quantities of CH_4_ that were stored in the anoxic hypolimnion can be effectively transported to the upper layer during rapid mixing, causing high dissolved CH_4_ concentrations in the surface water and diffusive fluxes to the atmosphere in the autumn [19]. Following the complete overturn, the entire water column could be oxic, with little of the original CH_4_ remaining in the water column [19], which influences the CH_4_ flux in the following season. In contrast, incomplete turnover is adverse to the emission of bottom CH_4_ and brings oxygen to only the middle layer, leaving the deep water anoxic with high CH_4_ storage [60]. Hence, the contribution of autumn emissions is largely determined by the length of the summer stagnation period and the intensity of turnover, and both processes are related to lake topography and morphology.

**Fig. 4:**
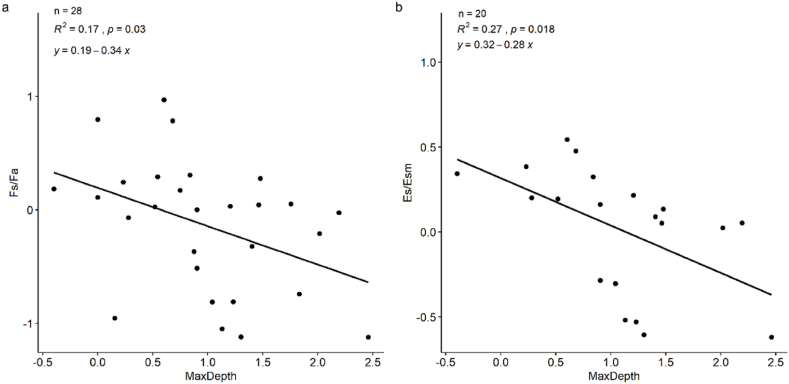
The ratio between CH_4_ flux in the summer and autumn (Fs/Fa) in relation to lake maximum depth (MaxDepth) (a) and the ratio of whole year CH_4_ emission estimation with flux in summer to that with seasonal mean flux (Es/Esm) in relation to lake maximum depth (MaxDepth) (b). Note that all the data were natural log transformed.

**Fig. 5:**
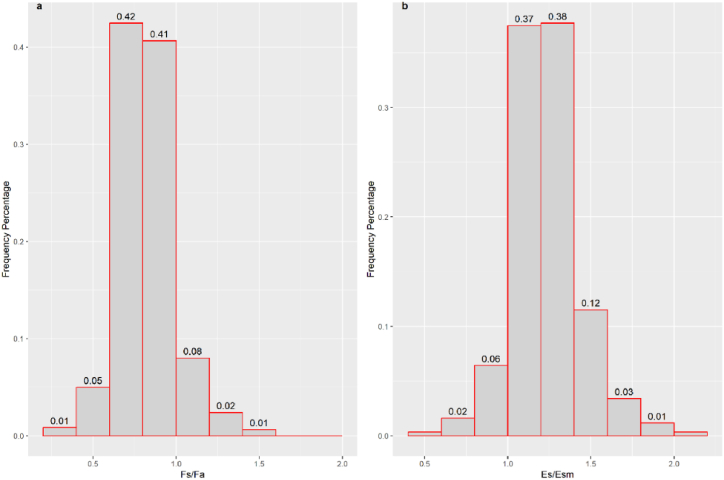
Frequency percentages of Fs/Fa and Es/Esm. Fs and Fa are the CH_4_ fluxes in the summer and autumn, respectively; Es represents the estimated whole-year CH_4_ emissions using the CH_4_ flux in the summer, and Esm represents the estimated CH_4_ emissions using the seasonal mean CH_4_ flux.

Limited by the data volume, only Esa/Esm and Fsummer/Fautumn showed significant correlations with maximum depth (Fig. 4a & b). We chose the linear regression models in Fig. 4 for the analysis of Fs/Fa and Es/Esm for global lakes in the mid-latitudes. The results showed that for global lakes with latitudes between 24 and 54, only 0.11% of lakes had higher CH_4_ fluxes in autumn than in summer (Fig. 5a), which is in accordance with the amount of relatively shallow lakes globally [61]. Similarly, less than 0.1% of lakes had Es/Esm <1, and 75% of lakes showed Es/Esm ranging from 1.0 to 1.4 (Fig. 5b), indicating that the summer flux is likely to overestimate the yearly CH_4_ emissions, but the error for most lakes with medium depths retains a low level (within 20%). Therefore, multi-season sampling should be implemented when lakes are relatively shallow or deep.

#### 3.2.3 High-latitude lakes

A total of 84 lakes with CH_4_ flux data during the ice-melt and ice-free seasons were included for the seasonal CH_4_ analysis. Due to the different methods used for CH_4_ flux measurement, 28 lakes included both CH_4_ diffusive and ebullitive fluxes, and 61 lakes had only CH_4_ diffusion data. Both CH_4_ total and diffusive fluxes showed significantly higher values in the ice-free period than during the ice-melt period (*p* < 0.005, Fig. 3c & d). For the 28 lakes with CH_4_ total flux, the contribution percentage of CH_4_ flux in the ice-melt period ranged from 0% to 56%, with a mean value of 17% and a medium value of 13%. In contrast, the CH_4_ diffusive flux of the 61 lakes showed ice-melt contribution percentages of 0%–83%, with mean and medium values of 30% and 21%, respectively. This result suggests that the ice-melt contribution could be overestimated when only the CH_4_ diffusive flux was used.

The CH_4_ total and diffusive fluxes in the ice-melt and ice-free periods all showed negative correlations with lake area and maximum depth (Table 1), indicating higher CH_4_ emissions in small and shallow lakes than in large and deep lakes. The CH_4_ diffusive flux showed more significant correlations to lake maximum depth and area than the CH_4_ total flux, probably suggesting that the CH_4_ diffusive flux is more related to lake morphology than the ebullitive flux. Nevertheless, the contribution of ice-melt CH_4_ total and diffusive flux showed no significant correlation with lake depth and area, and the correlation was relatively weak for CH_4_ diffusive flux compared to that of the CH_4_ total flux (Table 1). All the above results suggest the obvious influence of lake depth on CH_4_ ebullition during ice-off; in other words, shallow lakes tend to have higher CH_4_ ebullition in the ice-melt period than deep lakes, which may significantly improve their spring CH_4_ emission contribution.

**Table 1:** The relationships of CH_4_ fluxes in the ice-melt and ice-free periods with lake physical characteristics.

	**CH**_**4**_**total flux**			**CH**_**4**_**diffusive flux**		
	Ice-melt flux	Ice-free flux	Ice-melt Contribution	Ice-melt flux	Ice-free flux	Ice-melt Contribution
**Area**	−0.37	−0.34	−0.18	−0.59**	−0.51**	−0.10
**MaxDepth**	−0.43*	−0.32	−0.35	−0.50**	−0.62**	0.12
**Area/MaxDepth**	−0.19	−0.21	−0.040	−0.54**	−0.41**	−0.16

The data are Pearson correlation coefficients, * represents *p* < 0.05; ** represents *p* < 0.01.

The spring ice-off may produce significant CH_4_ emissions in high-latitude lakes after CH_4_ accumulates under the ice, which is dependent on the length of the ice-cover season, the availability of oxygen in the winter [25] and the ice-growth rate [62]. More importantly, the CH_4_ flux during spring ice-off is mostly determined by the intensity of lake turnover, which is usually complete in deep lakes [26]. Thus, we believe that the higher CH_4_ flux in the ice-free period is due to most of the shallow lakes in our data (only 7 out of the 28 lakes had maximum depths greater than 10 m), and the estimation of the CH_4_ flux contribution percentage in the ice-melt period should be conservative.

However, other factors may also result in the uncertainty of our estimation. A large majority of the northern lakes have been measured over short periods of only one to a few days, even in the ice-free season. Wik et al. (2016) [63] recommended that measurements of diffusive CH_4_ flux and ebullition be made over at least 11 and 39 days, respectively, and scattered throughout the ice-free season. The same problem also exists for studies during the ice-cover period. According to our statistics, the number of CH_4_ flux studies for high-latitude lakes in the ice-covered period (82) is much less than that in the ice-free season (150), and most studies in ice-covered lakes sampled only once or over a short time [64,65]. More importantly, a few studies have investigated spring emissions by calculating the difference between depth-integrated gas storage before the onset of ice thaw and after complete ice-off (e.g., Refs. [24,65,66]), which is termed potential spring emissions by Jansen et al. (2019) [25]. This estimation of spring emissions may have a large deviation since the possibility of CH_4_ oxidation during ice-off is not included [67]. In addition, ice breaks usually occur next to the shore first; when sampling after complete ice-off, the littoral zone may have accumulated CH_4_ in the bottom water, while the in situ CH_4_ efflux to the air may be similar to that of the profundal area [68]. Thus, spring CH_4_ emissions may be overestimated. Therefore, future research should elucidate the sampling strategy for CH_4_ flux studies in ice-covered lakes.

### 3.3 Limitations and uncertainties

The most obvious limitation in this study is the data size. For the study of spatial CH_4_ flux patterns, only 42 lakes were used, and 13 and 28 lakes were included for the seasonal CH_4_ flux analysis of the low- and mid-latitude lakes, respectively, which largely explained the relatively low R^2^ in the regression models. In fact, the spatial-temporal variability in lake CH_4_ flux has received much attention, especially since CH_4_ became a research hotspot. However, despite the various efforts that have been made, we found that the available data are still limited for our study objectives. For example, for spatial CH_4_ analysis, first, the lake area and depth must be clearly articulated in the literature, and some small lakes at low latitudes, such as the Pantanal region, Brazil, in Bastviken et al. (2010) [7], were excluded. Second, sampling should include both the littoral and profundal zones of a lake, and inner-zone spatial analysis data were excluded in our study.

The data size also limited a more precise analysis of spatial CH_4_ flux variability in different lake types. We tried a local weighted regression for L/P with lake morphology parameters and found varying forms of regression lines in different lake types. Then, we tried piecewise polynomial regression analysis and found revised U waves for lakes with log area <1 and log Area/MaxDepth <0 and U waves for the remaining part (Supplementary Fig. S1). While these relationships are not statistically significant thus far, more data are needed to examine their meaning and correction.

On the other hand, there is a lack of accurate quantitative standards for littoral, intermediate and profundal zones within lakes, and in this study, we hypothesized that these three zones were averaged in a lake. In fact, deep and shallow lakes should have different proportions of littoral and profundal zones; hence, for a shallow lake that usually had a higher flux and a larger littoral zone or a deep lake that had a higher flux and a larger profundal zone, our method would result in an underestimation of Em, and then, the El/Em for those shallow lakes and the Ep/Em for those deep lakes should be further decreased. However, the error of Em is uncertain for other lake types.

Overall, this study highlights several prospects for future work to reduce uncertainties. First, more available spatial and seasonal lake CH_4_ data are still urgently needed. Second, global research on lake morphology and topography should be strengthened to provide reasonable reference standards for the quantitative separation of different lake zones.

## 4 Conclusions

The spatial and seasonal variabilities in CH_4_ fluxes in lakes varying in latitude, area, and depth were analyzed at a global scale based on data from the literature. Spatially, most lakes showed higher CH_4_ fluxes in the littoral zone, except some small and deep lakes, and the flux differences between littoral and profundal zones increased with increasing lake area or area/max depth. Globally, half of the global lakes would have a relatively low error of CH_4_ emission estimation, while we emphasize the considerable spatial sampling design needed for large and deep lakes. Seasonally, more regional research is needed for low-latitude lakes due to their high variability in lake depth and area, which have different effects on CH_4_. The mid-latitude lakes showed higher CH_4_ fluxes in the summer and autumn, indicating the influence of temperature and autumn overturn, while the latter is related to the maximum depth. Globally, we pointed out the necessity of multi-season sampling in relatively shallower or deeper lakes. Generally, the high-latitude lakes showed significantly higher CH_4_ fluxes in the ice-free period than during the ice-melt period, while the deep lakes may be the opposite. This study is fundamental to elucidating the spatial and seasonal CH_4_ patterns for different lake styles and the sampling strategy for CH_4_ flux studies, which is beneficial for determining accurate CH_4_ budgets from global lakes.

## Author contribution statement

Lingling Li: Performed the experiments; Analyzed and interpreted the data; Contributed reagents, materials, analysis tools or data; Wrote the paper.

Bin Xue: Conceived and designed the experiments; Performed the experiments; Analyzed and interpreted the data.

## Data availability statement

Data will be made available on request.

## Declaration of competing interest

The authors declare that they have no known competing financial interests or personal relationships that could have appeared to influence the work reported in this paper.
