# Supplementary material for: Spatial-temporal variability of methane fluxes in lakes varying in latitude, area, and depth
Source: Heliyon. 2023 Jul 21;9(8):e18411. doi: 10.1016/j.heliyon.2023.e18411 (PMC10404696; doi:10.1016/j.heliyon.2023.e18411)
Supplement: Multimedia component 1 [file mmc1.docx]

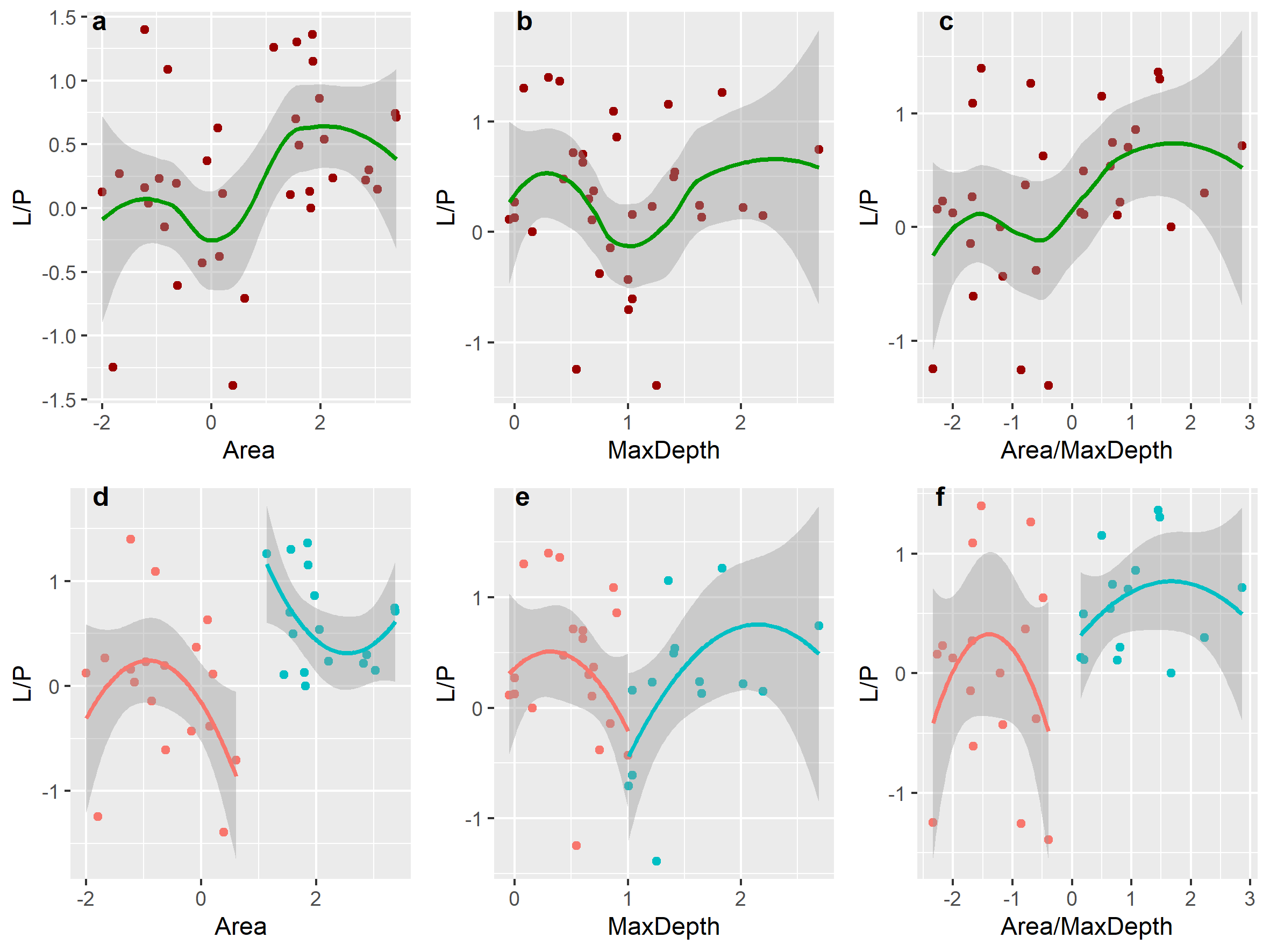


Figure S1 Locally weighted regression (upper) and piecewise polynomial regression (lower) analyses for L/P in relation with lake area (a & d), maximum depth (b & e), and Area/MaxDepth (c & f).

**Reference for Table S1**

Alvalá, P. C., and V. W. Kirchhoff. 2000. Methane fluxes from the Pantanal floodplain in Brazil: seasonal variation. Pages 95-99 in J. v. Ham, A. P. M. Baede, L. A. Meyer, and R. Ybema, editors. Non-CO2 Greenhouse Gases: Scientific Understanding, Control and Implementation. Springer, The Netherlands.

Bartosiewicz, M., I. Laurion, and S. MacIntyre. 2015. Greenhouse gas emission and storage in a small shallow lake. Hydrobiologia 757:101-115.

Bastviken, D., A. L. Santoro, H. Marotta, L. Q. Pinho, D. F. Calheiros, P. Crill, and A. Enrichprast. 2010. Methane Emissions from Pantanal, South America, during the Low Water Season: Toward More Comprehensive Sampling. Environmental science & technology 44:5450-5455.

Bergier, I., E. M. L. M. Novo, F. M. Ramos, E. A. Mazzi, and M. F. F. L. Rasera. 2011. Carbon Dioxide and Methane Fluxes in the Littoral Zone of a Tropical Savanna Reservoir (Corumbá, Brazil). Oecologia Australis 15:666-681.

Bohlin, V. and A. Anderö Nordqvist. 2021. Greenhouse gas emissions from three large lakes during the autumn 2020, Linköpings universitet.

Borges, A. V., G. Abril, B. Delille, J. P. Descy, and F. Darchambeau. 2011. Diffusive methane emissions to the atmosphere from Lake Kivu (Eastern Africa). Journal Of Geophysical Research-Biogeosciences 116:7.

Crill, P. M., K. B. Bartlett, J. O. Wilson, D. I. Sebacher, R. C. Harriss, J. M. Melack, S. MacIntyre, L. Lesack, and L. Smith‐Morrill. 1988. Tropospheric methane from an Amazonian floodplain lake. Journal of Geophysical Research: Atmospheres 93:1564-1570.

DelSontro, T., D. F. McGinnis, S. Sobek, I. Ostrovsky, and B. Wehrli. 2010. Extreme methane emissions from a Swiss hydropower reservoir: contribution from bubbling sediments. Environmental science & technology 44:2419-2425.

Devol, A. H., J. E. Richey, W. A. Clark, S. L. King, and L. A. Martinelli. 1988. Methane emissions to the troposphere from the Amazon floodplain. Journal of Geophysical Research: Atmospheres 93:1583-1592.

Devol, A. H., J. R. Richey, B. R. Forsberg, and L. A. Martinelli. 1990. Seasonal dynamics in methane emissions from the Amazon River floodplain to the troposphere. Journal of Geophysical Research Atmospheres 95:16417-16426.

Duan, X., X. Wang, Y. Mu, and Z. Ouyang. 2005. Seasonal and diurnal variations in methane emissions from Wuliangsu Lake in arid regions of China. Atmospheric Environment 39:4479-4487.

Fernández, J. E., F. Peeters, and H. Hofmann. 2014. Importance of the autumn overturn and anoxic conditions in the hypolimnion for the annual methane emissions from a temperate lake. Environmental science & technology 48:7297-7304.

Gorsky, A. L., N. R. Lottig, P. C. Stoy, A. R. Desai and H. A. Dugan. 2021. The Importance of Spring Mixing in Evaluating Carbon Dioxide and Methane Flux From a Small North‐Temperate Lake in Wisconsin, United States. Journal of Geophysical Research: Biogeosciences, 126(12).

Hallgren E and O. Åman. 2019. Methane fluxes in lakes at different spatiotemporal scales. Bachelor of Science, Linköpings universitet.

Hofmann, H. 2013. Spatiotemporal distribution patterns of dissolved methane in lakes: How accurate are the current estimations of the diffusive flux path? Geophysical Research Letters 40:2779-2784.

Iwata, H., K. Nakazawa, H. Sato, M. Itoh, Y. Miyabara, R. Hirata, Y. Takahashi, T. Tokida and R. Endo. 2020. Temporal and spatial variations in methane emissions from the littoral zone of a shallow mid-latitude lake with steady methane bubble emission areas. Agricultural and Forest Meteorology, 295: 108184.

Li, G., S. Zhang, X. Shi, L. Zhan, S. Zhao, B. Sun, Y. Liu, Z. Tian, Z. Li, L. Arvola, S. Uusheimo, T. Tulonen and J. Huotari. 2022. Spatiotemporal variability and diffusive emissions of greenhouse gas in a shallow eutrophic lake in Inner Mongolia, China. Ecological Indicators 145: 109578.

Liikanen, A., J. T. Huttunen, K. Valli, and P. J. Martikainen. 2002. Methane cycling in the sediment and water column of mid-boreal hyper-eutrophic Lake Kevätön, Finland. Archiv Fur Hydrobiologie 154:585-603.

Liu, R. M., A. Hofmann, F. O. Gulacar, P. Y. Favarger, and J. Dominik. 1996. Methane concentration profiles in a lake with a permanently anoxic hypolimnion (Lake Lugano, Switzerland-Italy). Chemical Geology 133:201-209.

Marani, L., and P. C. Alvalá. 2007. Methane emissions from lakes and floodplains in Pantanal, Brazil. Atmospheric Environment 41:1627-1633.

Marinho, C. C., C. Palma Silva, E. F. Albertoni, C. Trindade, and F. d. A. Esteves. 2009. Seasonal dynamics of methane in the water column of two subtropical lakes differing in trophic status. Brazilian Journal of Biology 69:281-287.

Martinez-Cruz, K., R. Gonzalez-Valencia, A. Sepulveda-Jauregui, F. Plascencia-Hernandez, Y. Belmonte-Izquierdo, and F. Thalasso. 2017. Methane emission from aquatic ecosystems of Mexico City. Aquatic Sciences 79:159-169.

Miao, Y., H. Meng, W. Luo, B. Li, H. Luo, Q. Deng, Y. Yao, Y. Shi and Q. L. Wu. 2022. Large alpine deep lake as a source of greenhouse gases: A case study on Lake Fuxian in Southwestern China. Sci Total Environ 838(Pt 2): 156059.

Miller, D. N., W. C. Ghiorse, and J. B. Yavitt. 1999. Seasonal patterns and controls on methane and carbon dioxide fluxes in forested swamp pools. Geomicrobiology Journal 16:325-331.

Miller, L. G., and R. S. Oremland. 1988. Methane efflux from the pelagic regions of four lakes. Global Biogeochemical Cycles 2:269-277.

Mosher, J. J., A. M. Fortner, J. R. Phillips, M. S. Bevelhimer, A. J. Stewart, and M. J. Troia. 2015. Spatial and Temporal Correlates of Greenhouse Gas Diffusion from a Hydropower Reservoir in the Southern United States. Water 7:5910-5927.

Murase, J., Y. Sakai, A. Sugimoto, K. Okubo, and M. Sakamoto. 2003. Sources of dissolved methane in Lake Biwa. Limnology 4:91-99.

Praetzel, L. S. E., M. Schmiedeskamp and K. H. Knorr. 2021. Temperature and sediment properties drive spatiotemporal variability of methane ebullition in a small and shallow temperate lake. Limnology and Oceanography.

Schilder, J., D. Bastviken, M. V. Hardenbroek, and O. Heiri. 2016. Spatiotemporal patterns in methane flux and gas transfer velocity at low wind speeds: Implications for upscaling studies on small lakes. Journal of Geophysical Research Biogeosciences 121:1456–1467.

Schubert, C. J., F. S. Lucas, E. Durisch-Kaiser, R. Stierli, T. Diem, O. Scheidegger, F. Vazquez, and B. Mueller. 2010. Oxidation and emission of methane in a monomictic lake (Rotsee, Switzerland). Aquatic Sciences 72:455-466.

Sepulveda-Jauregui, A., K. M. Walter Anthony, P. Anthony, G. Grosse, and J. Chanton. 2015. Methane and Carbon Dioxide Emissions from 40 Lakes Along a North-South Latitudinal Transect in Alaska. Biogeosciences 12:3197-3223.

Serikova, S., O. S. Pokrovsky, H. Laudon, I. V. Krickov, A. G. Lim, R. M. Manasypov, and J. Karlsson. 2019. High carbon emissions from thermokarst lakes of Western Siberia. Nature communications 10.

Smith, L. K., and W. M. Lewis Jr. 1992. Seasonality of methane emissions from five lakes and associated wetlands of the Colorado Rockies. Global Biogeochemical Cycles 6:323-338.

Tomelleri, E., K. Scholz, S. Pighini, F. Carotenuto, B. Gioli, F. Miglietta, R. Sommaruga, G. Tonon, A. Zaldei and G. Wohlfahrt (2023). A novel method for characterising the inter- and intra-lake variability of CH4 emissions: validation and application across a latitudinal transect in the Alpine region. Preprint.

Van Hardenbroek, M., A. F. Lotter, D. Bastviken, N. T. Duc, and O. Heiri. 2012. Relationship between δ13C of chironomid remains and methane flux in Swedish lakes. Freshwater Biology 57:166-177.

Walter, K. M., S. A. Zimov, J. P. Chanton, D. Verbyla, and F. S. Chapin, III. 2006. Methane bubbling from Siberian thaw lakes as a positive feedback to climate warming. Nature 443:71-75.

Wang, H., R. Huang, J. Li, Q. Chen and T. Ma. 2020. Dissolved and emitted methane in the Poyang Lake. Science China Technological Sciences, 64(1): 203-212.

Xiao, Q., M. Zhang, Z. Hu, Y. Gao, C. Hu, C. Liu, S. Liu, Z. Zhang, J. Zhao, and W. Xiao. 2017. Spatial variations of methane emission in a large shallow eutrophic lake in subtropical climate. Journal of Geophysical Research Biogeosciences 122.

Xing, Y. P., P. Xie, H. Yang, L. Y. Ni, Y. S. Wang, and K. W. Rong. 2005. Methane and carbon dioxide fluxes from a shallow hypereutrophic subtropical Lake in China. Atmospheric Environment 39:5532-5540.

Zhang, L., Q. J. H. Liao, R. Gao, R. Luo, C. Liu, J. C. Zhong and Z. D. Wang. 2021. Spatial variations in diffusive methane fluxes and the role of eutrophication in a subtropical shallow lake. Science of the Total Environment 759: 10.

Zhu, D., Y. Wu, H. Chen, Y. He, and N. Wu. 2016. Intense methane ebullition from open water area of a shallow peatland lake on the eastern Tibetan Plateau. Sci Total Environ 542:57-64.
